# Commentary: mobile laboratories for SARS-CoV-2 diagnostics: what Europe could learn from the East African Community to assure trade in times of border closures

**DOI:** 10.1186/s12992-021-00700-9

**Published:** 2021-04-23

**Authors:** Florian Gehre, Hakim Lagu, Emmanuel Achol, Michael Katende, Jürgen May, Muna Affara

**Affiliations:** 1 Department of Infectious Disease Epidemiology, Bernhard-Nocht-Institute for Tropical Medicine, Hamburg, Germany; 2 East African Community Secretariat, Health Department, Arusha, Tanzania; 3 Tropical Medicine II, University Medical Center Hamburg-Eppendorf (UKE), Hamburg, Germany; 4 German Center for Infection Research (DZIF), Hamburg-Borstel-Lübeck-Riems, Hamburg, Germany

**Keywords:** SARS-CoV-2 diagnostics, COVID-19, Pandemic, Border closures, Mobile laboratories, BSL4, East Africa, EAC, Electronic health certificate, Trade

## Abstract

**Background:**

The emergence of SARS-CoV-2 mutants might lead to European border closures, which impact on trade and result in serious economic losses. In April 2020, similar border closures were observed during the first SARS-CoV-2 wave in East Africa.

**Main body:**

Since 2017 the East African Community EAC together with the Bernhard-Nocht-Institute for Tropical Medicine BNITM established a mobile laboratory network integrated into the National Public Health Laboratories of the six Partner States for molecular diagnosis of viral haemorrhagic fevers and SARS-CoV-2. Since May 2020, the National Public Health Laboratories of Kenya, Rwanda, Burundi, Uganda and South Sudan deployed these mobile laboratories to their respective borders, issuing a newly developed “Electronic EAC COVID-19 Digital Certificate” to SARS-CoV-2 PCR-negative truck drivers, thus assuring regional trade.

**Conclusion:**

Considering the large financial damages of border closures, such a mobile laboratory network as demonstrated in East Africa is cost-effective, easy to implement and feasible. The East African Community mobile laboratory network could serve as a blueprint for Europe and other countries around the globe.

## Background

The corona virus pandemic is having a serious impact on public life and the economy of all European countries. The recent advent of new mutant strains, has led to travel restrictions for citizens of Europe. These travel restrictions not only impact on individual movements but also on trade.

## Main text

 The advent of the United Kingdom’s SARS-CoV-2 mutant B117 in December 2020, resulted in border closures between the United Kingdom and mainland Europe, leading to the build-up of an estimated 10.000 trucks at the ports of Dover alone [1]. Although France initially requested a negative PCR test as a condition for drivers to enter the country, it later accepted proof of a negative rapid diagnostic test (RDT), as the 24 h turn-around time for a PCR test was deemed too long, resulting in mounting queues of trucks [2]. The delay of each container caused economic damages for the freight forwarders and had knock-on effects on supply chains.

As more and more new mutants are likely to emerge, and as there are ever-changing differences in COVID-19 incidence between countries, scenarios in which even internal European borders could close again, are conceivable.

During April-June 2020, similar scenes of border closures were observed all over Africa [3]. In East Africa, pictures of long tailbacks of trucks at Ugandan-Kenyan or Kenyan-Tanzanian borders were broadcasted around the world in international news stations [3, 4]. These three countries, together with Burundi, Rwanda and South Sudan, are Partner States of an intergovernmental body, the East African Community (EAC, www.eac.int). Very similar to the European Union, the EAC established a customs union (which includes the health sector) and a common market with free movement of goods, labour, residence and capital, amongst others. A monetary union with a common currency is currently under development and once completed, shall lead to a political federation; the ultimate goal of the EAC regional integration.

In most EAC Partner States, prior to 2017, samples collected from periphery facilities had to be transported to the central National Public Health Laboratories (NPHL) for PCR diagnosis, often resulting in significant delays in turn-around-times in excess of 72 h. However, between 2017 and 2020, the EAC Secretariat, with funding from the German Ministry for Economic Cooperation and Development (BMZ) through the German Development Bank (KfW) [5], and together with the Bernhard-Nocht-Institute for Tropical Medicine (BNITM), set up a regional network of nine mobile laboratories. These laboratories are capable of diagnosing not only haemorrhagic viruses, but also SARS-CoV-2. Technical laboratory staff were trained in PCR and ELISA and the laboratories were handed over to the Partner States’ Ministries of Health. With a basic setup of one centrifuge (24 tube rotor) and two PCR machines, 4–6 trained laboratory staff can process around 400 samples per shift. Centrifuges (an identified bottleneck for RNA extraction) and PCR machines (for SARS-CoV-2 diagnosis) can easily be added to upgrade the mobile laboratories, and processing samples in several shifts could thus increase throughput and sample turnover, according to the local needs. Within the project, SARS-CoV-2 diagnostic tests satisfactorily validated by the Foundation for Innovative Diagnostics (FIND) were utilised in the mobile labs. However, Partner States also received donations of diagnostic tests from other funders.

**Fig. 1:**
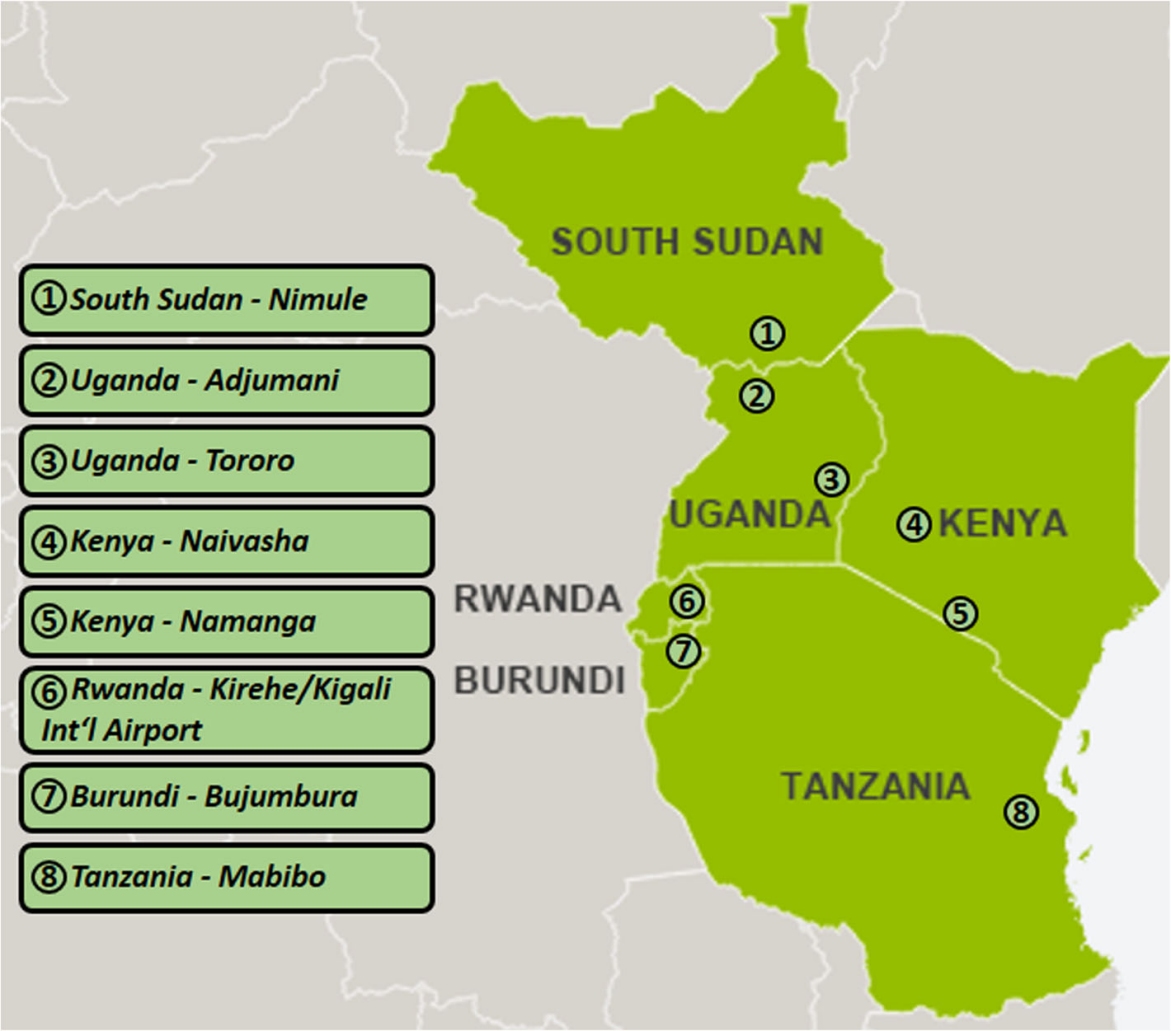
Map showing the deployment locations of the SARS-CoV-2 diagnostic mobile laboratories along strategic roads and internal borders of the East African Community

With a turn-around-time of 8 h for SARS-CoV-2 diagnosis, five countries in the region (Uganda, Kenya, Rwanda, South Sudan and Burundi) decided in May/June 2020 to deploy these laboratories to their respective borders with the mandate to clear the backlog of trucks, thus assuring regional trade ever since (see Fig. 1). Up to February 2021, seven mobile laboratories, operated by trained NPHL personnel, have been responsible for processing 280.728 samples (most of which from truck drivers and travellers) in remote border areas throughout the East African region.

**Fig. 2:**
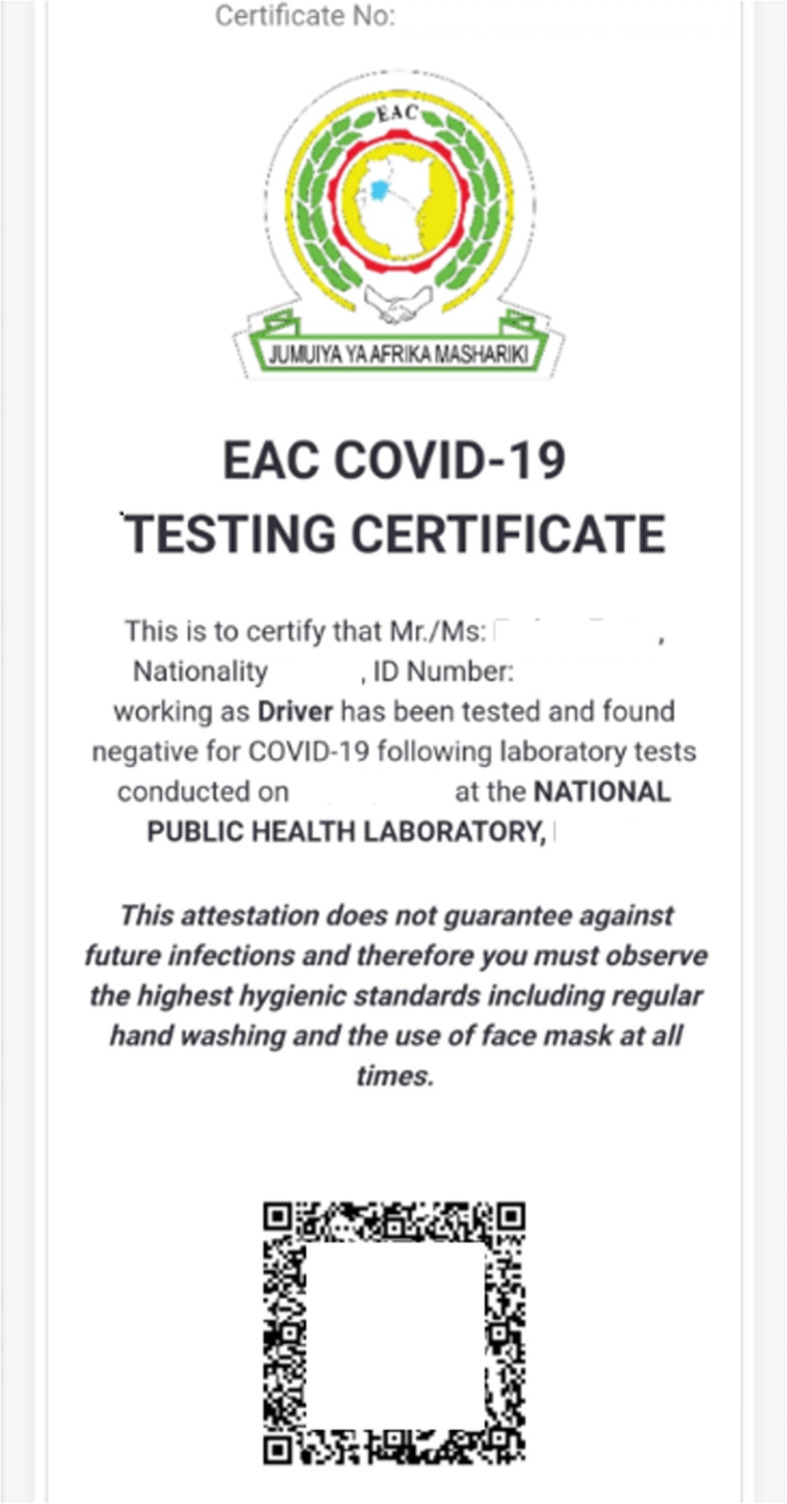
The electronic “East African Community COVID-19 testing certificate”. The certificate allows SARS-CoV-2 PCR negative truck drivers to move freely for 2 weeks within the region and transport freight between countries. It is numbered and captures name, nationality, ID/passport number, and the country and designation of the testing laboratory. All information is also captured in a machine readable QR-code for quick processing by health authorities, checkpoints and law enforcement officers

This was further facilitated by the development of the Regional “EAC Electronic Cargo and Drivers Tracking system”[6], which allowed tracking of the “Electronic EAC COVID-19 Digital Certificate” for easy identification and clearing of truck drivers who tested negative for the SARS-CoV-2 virus. Truck drivers need to download the app, register in the system and after confirmation of a negative SARS-CoV-2 PCR test result, a numbered certificate is electronically issued capturing the driver’s name, nationality, ID number, date of testing and the issuing laboratory and country (see Fig. 2). This certificate will then serve as proof for further travels at checkpoints, health authorities and for law enforcement officers and is recognised within the entire EAC region. Although the certificate is valid for 14 days, drivers are obliged to obtain a new certificate in case of emerging symptoms.

Once validated that negative rapid lateral flow tests can serve as a predictor for SARS-CoV-2 transmission, these can easily be included in the diagnostic workflow of the mobile laboratories – until then, however, PCR as the gold standard is used in our set-up to assess infectiousness of drivers and prevent cross-border transmission.

Although positivity rates are sensitive data for most countries, when reported, we observed between 3 and 6 % of positive PCR results from some of the laboratories. Each driver that tests positive will receive counselling through public health officials at the testing locations, and will then be advised to follow respective country guidelines, such as seeking medical assistance or moving into home quarantine. In case of non-residents, entry will be refused into the country of destination.

## Conclusion

 With the current project, we demonstrated that by simply combining a rapidly developed regional electronic health certificate tracking system with laboratory services, the added value of the EAC mobile laboratory network was significantly extended from mere patient diagnostics to maintaining regional trade.

This kept the East African economy functional and guaranteed the exchange of goods (including food supplies, medical goods, etc.) across borders. The continued transport of goods was especially important for landlocked countries such as Burundi, Rwanda, Uganda and South Sudan. Stationing the laboratories at points-of-entry, thus identifying positive patients early, will also have an effect on reducing the spread of epidemics across borders. In the future, lab deployment into outbreaks could also be guided by novel predictors of SARS-CoV-2 transmission hotspots and fatality rates [7].

The laboratories are coordinated through the countries’ NPHLs and therefore integrated in the national pandemic preparedness programs, with access to donor funding, making them a sustainable investment. In addition, many countries trained local laboratory experts originating from border areas to assure operations.

However, in geographically remote areas where whole laboratory teams need to be sent, long field deployments are not only expensive, but also a burden on the laboratory personnel of the NPHL. Another limitation is that supply of laboratory consumables and test kits relies, to a certain degree, on outside donors, which can interrupt laboratory work flows.

Taken together, our project has shown the importance of screening at point-of-entries during epidemics, and demonstrates how utilizing mobile laboratories in border regions (as exemplified during the SARS-CoV-2 pandemic), significantly impacts on the regional pandemic response and should be a considered strategy in future outbreaks. In particular, the new aspect of facilitating cross border trade by clearing SARS-CoV-2 negative truck drivers is not only an underestimated novel added value of mobile laboratories, but also a lesson that other regions could learn from the East African Community.

## Data Availability

Not applicable.

## Abbreviations

EAC: East African Community
BNITM: Bernhard-Nocht-Institute for Tropical Medicine
NPHL: National Public Health Laboratory
BMZ: Bundesministerium für wirtschaftliche Zusammenarbeit und Entwicklung
KfW: Kreditanstalt für Wiederaufbau
SARS-CoV-2: Severe acute respiratory syndrome-Corona Virus-2
COVID-19: Corona Virus Disease-2019
PCR: Polymerase Chain Reaction
ELISA: Enzyme linked Immuno-Sorbent Assay
